# Ultrathin materials for wide bandwidth laser ultrasound generation: titanium dioxide nanoparticle films with adsorbed dye†

**DOI:** 10.1039/d3na00451a

**Published:** 2023-07-17

**Authors:** Tiago B. Pinto, Sara M. A. Pinto, Ana P. Piedade, Carlos Serpa

**Affiliations:** a CQC-IMS, Department of Chemistry, University of Coimbra 3004-535 Coimbra Portugal serpasoa@ci.uc.pt; b CEMMPRE, Department of Mechanical Engineering, University of Coimbra 3030-788 Coimbra Portugal

## Abstract

Materials that convert the energy of a laser pulse into heat can generate a photoacoustic wave through thermoelastic expansion with characteristics suitable for improved sensing, imaging, or biological membrane permeation. The present work involves the production and characterization of materials composed of an ultrathin layer of titanium dioxide (<5 μm), where a strong absorber molecule capable of very efficiently converting light into heat (5,10,15,20-tetrakis(4-sulfonylphenyl)porphyrin manganese(iii) acetate) is adsorbed. The influence of the thickness of the TiO_2_ layer and the duration of the laser pulse on the generation of photoacoustic waves was studied. Strong absorption in a thin layer enables bandwidths of ∼130 MHz at −6 dB with nanosecond pulse laser excitation. Bandwidths of ∼150 MHz at −6 dB were measured with picosecond pulse laser excitation. Absolute pressures reaching 0.9 MPa under very low energy fluences of 10 mJ cm^−2^ enabled steep stress gradients of 0.19 MPa ns^−1^. A wide bandwidth is achieved and upper high-frequency limits of ∼170 MHz (at −6 dB) are reached by combining short laser pulses and ultrathin absorbing layers.

## Abstract 2

Strong absorption in a thin nanostructured layer enables photoacoustic generation of wide ultrasound bandwidths with nanosecond/picosecond lasers, improving imaging, sensing, or interaction with biological membranes.

## Graphical abstract

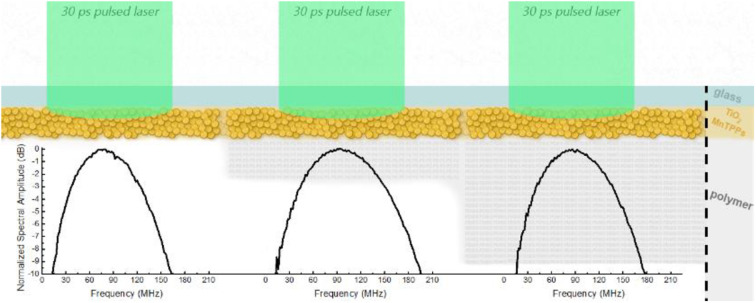

## Introduction

Thin materials with optimized optical and thermoelastic properties enable the production of intense and high-frequency ultrasound pulses through the photoacoustic effect. Those ultrasound pulses can be used in diversified areas, such as medical treatments (therapeutic ultrasound), material chemistry, biomedicine, ultrasound metrology, or engineering applications.^1–7^ Partially motivated by the application in diagnostic ultrasound imaging, where higher frequency components are required to improve image resolution, over the last two decades the research for new materials capable of converting light into pressure waves reaching high central frequencies and wide bandwidths has intensified.^8,9^ The generation of ultrasound waves through the photoacoustic effect consists of the absorption of a short laser pulse, with moderate optical energy fluence (typically <100 mJ cm^−2^), by an optically absorbing material which optimally should convert all absorbed light into heat within the laser pulse duration. When within the material a molecule can be identified as the light absorber, the laser pulse promotes the transition to a transient molecular excited state. Release of heat to the surroundings is enabled by the fast return to the ground state through non-radiative processes. This causes a transient thermoelastic expansion and launches a longitudinal pressure wave that propagates in the material at the speed of sound, with a moderate increase in the pressure of the absorbing medium. Such acoustic waves are characterized by pressure amplitudes over 1 MPa and wide bandwidths (that can reach ∼80 MHz at −6 dB).^6,10^ These materials are called piezophotonic or optoacoustic materials^11^ and they require a high linear absorption coefficient (*μ*_a_) in order to absorb a large amount of light in a thin layer, ultrafast radiationless transitions and a high Grüneisen coefficient, which reflects the behavior of the volume expansion as a function of an increase in the temperature. High-frequency ultrasound generated by piezophotonic materials has been applied in high-resolution imaging,^12^ inspection of materials,^13^ metrology,^14,15^ real-time analytical processes,^16^ or permeabilization of biological barriers.^11,17,18^

Piezophotonic materials generally comprise a thin film of the absorbing molecules or particles embedded on a thermal expansion polymer. Polystyrene (PS) and polydimethylsiloxane (PMDS) are commonly used as the thermal expansion layer due to their high thermal expansion coefficient. The full polymer layer is usually thicker than the actual absorption layer, as the latter layer is often obtained through nanomaterials grown on a glass substrate, which are then covered by a polymer layer. The intensity and shape of the ultrasound pulses also depend on the existence of rigid boundaries. Absorber materials consisting of carbon nanoparticles are frequently used, due to their capability to absorb linearly the incident light in a large range of wavelengths, and due to the nanometric scale, which allows the rapid and efficient deposition of thermal energy in the material which will suffer the expansion. Carbon black,^19^ carbon nanotubes,^20,21^ carbon nanofibers,^22^ reduced graphene oxide^23^ and graphene^24^ or candle soot nanoparticles^25^ are some of the commonly used materials. Other absorbers can also be used, such as dyes embedded in polystyrene,^11^ metallic films, gold nanostructures or nanoparticles.^26,27^ Carbon nanotubes functionalized with siloxane groups have been shown to generate exceptionally wide bandwidths (170 MHz at −6 dB) and peak pressures >1 MPa when excited by picosecond pulsed lasers.^10^

An efficient photoacoustic wave generation must comprise two conditions defined as thermal and stress (or optical) confinement. Thermal confinement requires total heat deposition before heat diffuses through the material, which maximizes the temperature in the irradiated region.^28,29^ It can be expressed as *τ*_L_ < *τ*_th_, where *τ*_th_ = (*μ*_a_^2^ × *α*_th_)^−1^ is the time of thermal diffusion (*α*_th_/m s^−1^ is the thermal diffusivity) and *τ*_L_ the laser pulse duration (FWHM, Full Width at Half Maximum). On the other hand, the stress confinement requires the heating of the irradiated region to occur before the thermal expansion takes place, *i.e.*, *τ*_L_ < *τ*_s_, where *τ*_s_ = (*μ*_a_ × *c*_s_)^−1^ is the time of the thermal expansion (*c*_s_/m s−^1^ is the speed of sound in the material). When both conditions are met, a situation known as the short-pulse regime, the volume expansion during the optical heating is negligible, *i.e.*, d*V*/*V* ≈ 0.^10^ In this situation, the relation between the pressure, *p*_0_, and temperature, Δ*T*, is given by^30^1
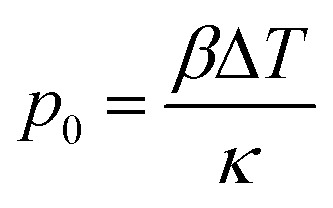
where *β* is the thermal coefficient of volume expansion (K^−1^), *κ* = *C*_P_/*ρc*_s_^2^*C*_V_ is the isothermal compressibility (Pa^−1^), *ρ* is the density (g dm^−3^), and *C*_P_ and *C*_V_ are the calorific capacities at constant pressure and volume, respectively. In solid materials, *C*_P_ and *C*_V_ are identical.^31^ Furthermore, the variation in temperature can be defined as Δ*T* = *H*_th_/*ρC*_P_*V*, where *H*_th_ = *E* × *A* is the energy converted into heat (J), assuming total conversion of the light absorption, *E* is the optical energy, *A* the absorption and *V* is the irradiated volume (m^3^). As mentioned previously, efficient photoacoustic wave generation requires the conversion of the total optical energy absorbed into heat (*η*_th_ ≈ 1) and an efficient thermal expansion as a response to an increase of the temperature, described by the dimensionless Grüneisen coefficient *Γ* = *β*/*κρC*_P_. So, the pressure equation simplifies to2
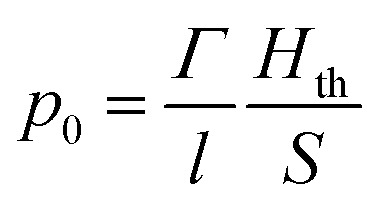
where *l* is the thickness of the absorbing region (m) and *S* is the illuminated area (m^2^). The expression above, highlighting the inversely proportional effect of the thickness, can be written as a similar equation using the earlier-mentioned linear absorption coefficient (*μ*_a_):^32^

3 *p*_0_ = *Γ* × *μ*_a_ × *F* × *η*_th_

where *F* is the energy fluence (J cm^−2^). Following eqn (3) in the short-pulse regime the photoacoustic wave amplitude depends on the intrinsic properties of piezophotonic materials and laser pulse characteristics. It requires a high thermal expansion and low heat capacity. Furthermore, it predicts that, for the same amount of optical energy absorbed, *p*_0_ will be greater the smaller the thickness. Absorption and expansion in an ultrathin layer launch an expansion in a short volume, enhancing the photoacoustic wave intensity and absolute pressures obtained. Moreover, if the thickness of the film absorption layer is much larger than the light penetration depth, this will cause acoustic attenuation (both in intensity and in bandwidth, at the cost of high frequencies). There is a reciprocal relation between having a very thin film (large *μ*_a_) required for the generation of broad bandwidths and the short-pulse regime condition, since it can turn *τ*_L_ < *τ*_s_ into a false inequality. The width of a photoacoustic wave is given by *τ*_L_ + 1/*c*_s_*μ*_a_, where 1/*c*_s_*μ*_a_ is the time that the perturbation takes to pass through the optical absorption length.^5^ The short-pulse regime is predominant in thicker films, where 1/*μ*_a_ ≫ *cτ*_L_ and the photoacoustic generation is given by eqn (3). On the other hand, if the *μ*_a_ is too large as in very thin films, the optical penetration depth becomes smaller than the pulse duration, *i.e. cτ*_L_ ≫ 1/*μ*_a_, and it is defined as long-pulse regime, expressed as4
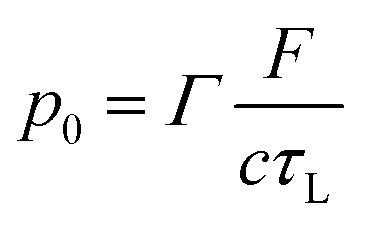


Under these conditions, the temporal profile of the photoacoustic pulse is limited by the laser pulse width. The use of shorter laser pulses leads to higher pressure amplitudes. Furthermore, it can revert the system to the short-pulse regime, since *τ*_L_ < *τ*_s_ becomes true.^10^

Herein we propose a method for production of ultrathin piezophotonic materials based on a mesoporous titanium dioxide layer with an adsorbed dye, embedded and covered with silicone paste, PS or PDMS to enable efficient thermoelastic expansion, capable of generating high frequency and broad bandwidth ultrasound. We used a picosecond laser to overcome the limitations associated with the generation of photoacoustic waves in the long-pulse regime, which is commonly seen in films with very low thicknesses when nanosecond laser pulses are used.

## Experimental

### Synthesis

The molecule 5,10,15,20-tetrakis(4-sulfonylphenyl)porphyrin manganese(iii) acetate (MnTPPS) was synthesized using the process reported in the literature.^33^ MnTPPS was synthesized by the condensation of pyrrole with benzaldehyde (7 : 3), followed by chlorosulphonation with addition of an excess of chlorosulfonic acid to TPP which ends with a hydrolysis. The Mn^III^ was added as an excess of tetrahydrate manganese acetate in sodium acetate/acetic acid and purified by size exclusion column chromatography (see the ESI† for details).

### Fabrication of piezophotonic materials

For TiO_2_ films production we used a Ti-nanoxide HT/SP paste (Solaronix), ideal for screen-printing, which allows a porous thin layer of particles with individual size between 10 and 15 nm to be obtained. The TiO_2_ colloidal paste is applied over a porous screen and the content is dragged with a rubber material to spread over a glass slide. The transparent TiO_2_ film was left to dry at room temperature and then subjected to a sintering process with a temperature program in an oven (Harry Gestigkeit Programme Regulator 5; 125 °C for 15 min, 325 °C for 10 min, 375 °C for 15 min, 450 °C for 15 min and 500 °C for 20 min). Films prepared by this method had thickness between 3.5 and 5.3 μm (measured first using a caliper and confirmed by electronic microscopy images). Casting using a doctor blade technique allowed films with thickness between 5.5 and 7.0 μm to be prepared. The prepared TiO_2_ films were then left to adsorb the dye in a concentrated solution of MnTPPS in ethanol, by submersing the substrate in the solution for certain time periods depending on the absorbance required (see absorbance values in Table 1). First, films with thicknesses between 3.5 and 7.0 mm were examined. For further detailed studies, we choose the thinner films that could reach an absorbance of 1.0 at 471 nm; those were the films with a TiO_2_ thickness of 4.4 μm. Full ultrasound characterization, measurements of absolute pressures reached, and laser-induced threshold studies were performed with those thin films.

**Table 1:** Properties of piezophotonic materials: absorbance (*A*) at 471 and 532 nm^a^

Film	Wavelength [nm]	*A*	*μ*_a_ a [mm^−1^]	6 ns	6 ns		30 ps	
				Peak pressure [MPa]	Central frequency	−6 dB bandwidth [MHz] (upper limit)	Central frequency	−6 dB bandwidth [MHz] (upper limit)
TiO_2__MnTPPS (silicone paste)	471	1.0	523	0.92	72	113 (134)	—	—
	532	0.26	136	—	58	82 (99)	79	121 (143)
TiO_2__MnTPPS_PS	471	1.0	523	0.49	82	133 (157)	—	—
	532	0.26	136	—	68	108 (127)	96	148 (173)
TiO_2__MnTPPS_PDMS	471	1.0	523	0.59	88	133 (160)	—	—
	532	0.26	136	—	74	105 (127)	87	131 (157)

a *μ*_a_ = 2.3*A*/*l*, Naperian absorption coefficient for each wavelength, where *l* = 4.4 μm; obtained peak pressures, central frequency, bandwidths at −6 dB and (in brackets) the upper-frequency limit at −6 dB, for pulse widths of 6 ns (10 mJ cm^−2^) and 30 ps (2 mJ cm^−2^)

The thin TiO_2_ films were covered with silicone paste, polystyrene or polydimethylsiloxane. To prepare polystyrene, 2 g of polymer were dissolved in 6 ml of toluene and left in a hot bath at 50 °C with constant stirring for 2 h, until a homogeneous solution could be seen. We used a closed recipient to avoid solvent evaporation and consequent solidification of the polymer. The preparation of polydimethylsiloxane involved the addition of a crosslinking agent to the PDMS monomer (1 : 9), with slight agitation for 5 minutes. The solution was then placed in an ultrasound bath at room temperature in a closed container.

Both polymers were added to the TiO_2_ films with MnTPPS adsorbed using a spin coater (Specialty Coating System, Inc., Model P6700). We used a rotation program, previously optimized, with 400 rpm for 10 s, to ensure the infiltration on the structure of TiO_2_, followed by 3000 rpm for 80 s. Due to their distinct viscosities by using this procedure a layer of 5 μm of PS and a layer of 15 μm of PDMS over the TiO_2_ film is obtained. The films were left at room temperature to evaporate the solvent. For those films with PDMS it was necessary to use a vacuum system in order to remove unwanted air bubbles.

### Ultrasound characterization

**Fig. 1:**
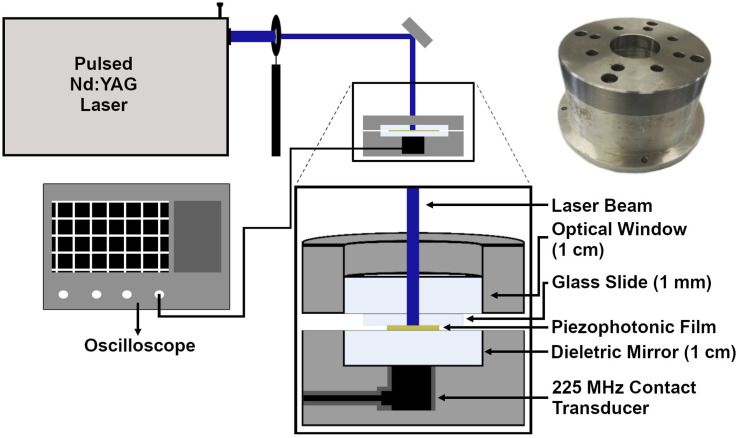
Setup used to measure high frequencies and bandwidths using a 225 MHz transducer; laser beam spot with a 2 mm diameter. (Top right) Photograph of the piezophotonic materials and transducer holder.

The photoacoustic waves were generated with a pulsed 6 ns FWHM Nd:YAG laser (EKSPLA NL301G NdYAG) that when necessary was coupled with an OPO (EKSPLA OPO PG-122) or with a 30 ps FWHM Nd:YAG laser (EKSPLA 2143A), with a diameter of 2 mm. With the ns pulse laser, a fluence of 10 mJ cm^−2^ was used, while with the ps laser, a fluence of 2 mJ cm^−2^ was used. The presence of high frequencies was analyzed using a front-face irradiation setup (Fig. 1) developed by our group for photoacoustic calorimetry experiments,^29^ with a 225 MHz contact transducer (Panametrics/Olympus, model V2113). We used a quartz mirror capable of reflecting >99% of the incident light to ensure the safety of the detector. It must be noted that the results were obtained using this quartz mirror of 1 cm between the material to be analyzed and the transducer, so high frequencies may be significantly attenuated. The piezophotonic material was placed on top of the mirror, which is inside of a support, and we applied silicon or water gel to improve the acoustic coupling. Finally, a heavy material (1.5 kg) with an optical window was placed over the film to ensure confinement. The signal was recorded using an oscilloscope (DPO7254 Tektronix, 2.5 GHz bandwidth) with an average of at least 200 waveforms. Films were replaced whenever they appeared to be damaged.

### Absolute pressures

**Fig. 2:**
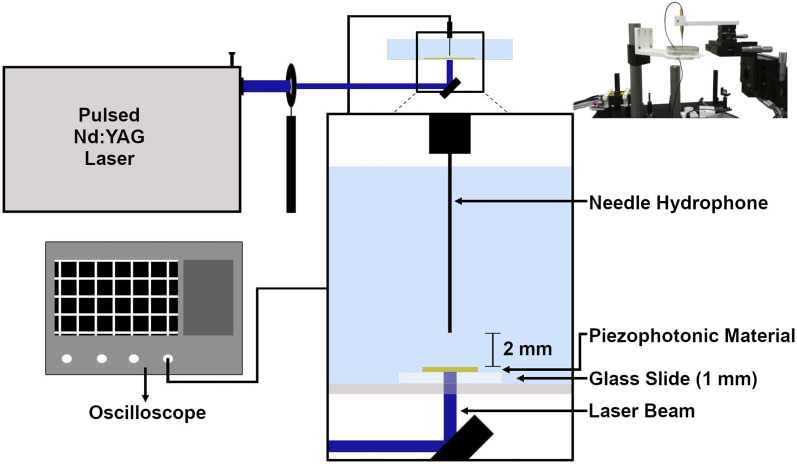
Setup used to measure absolute pressures with a 30 MHz needle hydrophone; laser beam spot with a 2 mm diameter. (Top right) Photograph of the setup.

Absolute pressure measurements were made using a 0.2 mm needle hydrophone (Precision Acoustics, model NH0200) calibrated in a range of 1 to 30 MHz. The photoacoustic waves were generated employing a nanosecond Nd:YAG laser (EKSPLA OPO PG-122 pumped by a EKSPLA NL301G Nd:YAG laser with pulse duration of 6 ns), with excitation at 471 nm and with a diameter of 2 mm, yielding a fluence of 10 mJ cm^−2^. The piezophotonic materials were placed in a water container at room temperature and the hydrophone was submersed until reaching a distance of 2 mm from the film (Fig. 2). Before placing the piezophotonic materials and positioning the hydrophone at the measuring distance, the laser beam was collinearly aligned with the tip of the hydrophone. The signal was recorded in an oscilloscope (DPO7254 Tektronix, 2.5 GHz bandwidth) with an average of at least 200 waveforms. To obtain the absolute pressure we used the recommendations and calibration provided by the manufacturer in a range between 1 and 30 MHz. A sampling interval of 2.5 × 10^11^ s was used to measure the FFT of the waveforms with the hydrophone and obtain their frequency distributions. The contribution of each frequency, with a step of 1 MHz from 0 to 30 MHz, was calculated to obtain the total distribution of frequencies for each waveform. A final calibration factor was obtained from the calibration factors provided by the hydrophone manufacturer for the various frequencies. Calibration factors of each waveform were employed to convert the measured pressure wave to the corresponding absolute pressure in MPa.

### Laser damage thresholds and performance under continuous laser exposure

The piezophotonic materials' robustness was quantified in terms of the observable laser-induced damage threshold and in terms of performance under continuous laser exposure. Under identical irradiation conditions, we compared the laser-induced damage of films with absorbance of 0.26 at 532 nm. A 30 ps FWHM Nd:YAG laser (EKSPLA 2143A) at 532 nm with an 8 mm diameter was used. We followed the measurement steps by increasing the incident laser fluence (between 20 mJ cm^−2^ and 120 mJ cm^−2^) and observing possible physical damage between steps. A digital camara was used to capture optical images of the films. Using the same laser source, the films were examined under continuous laser exposure (20 mJ cm^−2^, 10 Hz) for a period of one hour. Before irradiation and every 15 minutes after the start of irradiation the photoacoustic waves were measured by a 225 MHz contact transducer (Panametrics/Olympus, model V2113) using the same setup described above for ultrasound characterization.

## Results

### Properties of piezophotonic materials

Titanium dioxide (TiO_2_) films can be easily fabricated from a paste of nanoscale TiO_2_ particles, resulting in very thin (<8 μm in the present work) transparent films. Furthermore, they consist of a structure capable of adsorbing a large number of molecules due to the large surface area of the nanoscale particles. In this work, we use as absorbing dye 5,10,15,20-tetrakis(4-sulfonylphenyl)porphyrin manganese(iii) acetate (MnTPPS), a metalloporphyrin with a manganese(iii) atom that ensures the total conversion of the light absorbed into heat through deactivation of the excited state by radiationless processes. This happens because the half-filled orbitals of the metal are between the energy levels of the porphyrin HOMO and LUMO orbitals.^34^ This offers an alternative path to return to the ground state after excitation by charge transfer states from ligand-to-metal or metal-to-ligand, resulting in lifetimes lower than 0.5 ps in the excited state.^35,36^ Sulfonyl groups in the dye act as an anchor and allow the strong adsorption of the dye to the TiO_2_ nanoparticles. In addition, we incorporate silicone paste, polystyrene or polydimethylsiloxane into the TiO_2_ mesoporous films to optimize thermal expansion after the heat deposition and ensure a film protection layer. The mesoporous nature of the TiO_2_ films allows the incorporation of the polymers.

The piezophotonic materials were produced with thicknesses between 3.5 and 7.0 μm. By controlling the time of adsorption the absorbance was found to be 0.6 at 471 nm, which corresponds to the Soret band of MnTPPS. Polystyrene and polydimethylsiloxane permeated and covered the TiO_2__MnTPPS films. A third film was studied without adding polymer, but we used silicone paste interpenetrated into the TiO_2_ nanoparticles as expanding medium. The methodology to produce TiO_2_ films was optimized in order to improve the photoacoustic performance of these materials. The first step was to analyze the influence of the thickness in the generation of photoacoustic waves considering eqn (2). As expected, we observed that the intensity of the photoacoustic signal increases with the reciprocal of thickness (Fig. S3, ESI†). Also, thinner TiO_2__MnTPPS films (impregnated with silicone paste) exhibit higher central frequencies and broader bandwidths than thicker films. By doubling the film's thickness from 3.5 to 7.0 μm the central frequency changes from 56 to 38 MHz and the maximum bandwidth frequency at −6 dB diminishes from 115 to 65 MHz (Fig S4, ESI†). For further studies, we chose a film that was as thin as possible (4.4 μm), but it absorbed at least 90% of the incident light. By doing so we aim not to compromise on obtaining high frequencies, while still retaining high absorption, not compromising on the photoacoustic wave amplitude when irradiating at the maximum absorption wavelength.

**Fig. 3:**
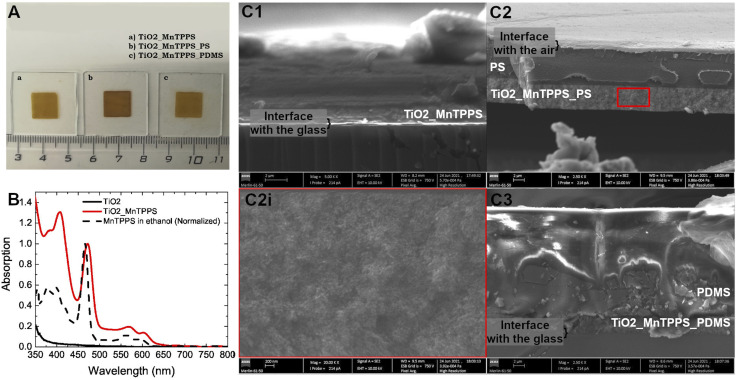
(A) Representative macrographs of the studied piezophotonic materials: (a) TiO_2__MnTPPS; (b) TiO_2__MnTPPS_PS; (c) TiO_2__MnTPPS_PDMS; (B) absorption spectra of the studied piezophotonic films; the dashed line represents the spectrum of MnTPPS in ethanol; (C) SEM macrographs of piezophotonic materials (cross-section): (C1) TiO_2__MnTPPS; (C2) TiO_2__MnTPPS_PS and (C2i) magnification of the TiO_2_ region in (C2); (C3) TiO_2__MnTPPS_PDMS.

Fig. 3A shows the representative photographs for each TiO_2_ material studied (with an absorbing layer of 4.4 μm). The nomenclature attributed to each film is based on their composition, *i.e.*, first the TiO_2_ substrate, then the MnTPPS dye and finally the polymer used (PS or PDMS). Fig. 3B shows the absorption spectrum of MnTPPS adsorbed on TiO_2_ (with an absorbance of 1.0 at 471 nm), as well as MnTPPS in an ethanol solution (the absorption spectrum was normalized for the sake of simplicity). Furthermore, we also recorded the TiO_2_ absorption spectrum without any dye adsorbed, showing no absorption in the visible range. The thickness and composition of each material were studied by scanning electron microscopy (Fig. 3C). TiO_2_ nanoparticles appear to be interpenetrated by the polymers. The relative volume of the polymer is higher than that of the ceramic nanoparticles. These are the reasons why in Fig. 3 there seems to exist two layers: the bottom one with the denser ceramic material interpenetrated with the polymer, and the outermost one with the polymer. The higher resolution micrograph, Fig. 3(C2i), shows an image with low particle boundary resolution due to the presence of the polymer. These images confirm that the thickness of the absorption region of the films corresponds to 4.4 μm. With matched absorption of 1, the materials proposed here have a high value of linear absorption coefficient of 523 mm^−1^. Table 1 presents the properties of each material studied, each absorption at 471 and 532 nm, and the respective linear absorption coefficient for both wavelengths. In addition, we report the values of pressure, central frequency, and bandwidths at −6 dB obtained experimentally. The bandwidth is defined by the difference between the upper and lower frequency limits measured at the amplitude of −6 dB. The high-frequency ultrasound transducer used underestimates the low-frequency region. The upper limit frequency obtained at −6 dB is also shown in Table 1.

### Ultrasound performance

**Fig. 4:**
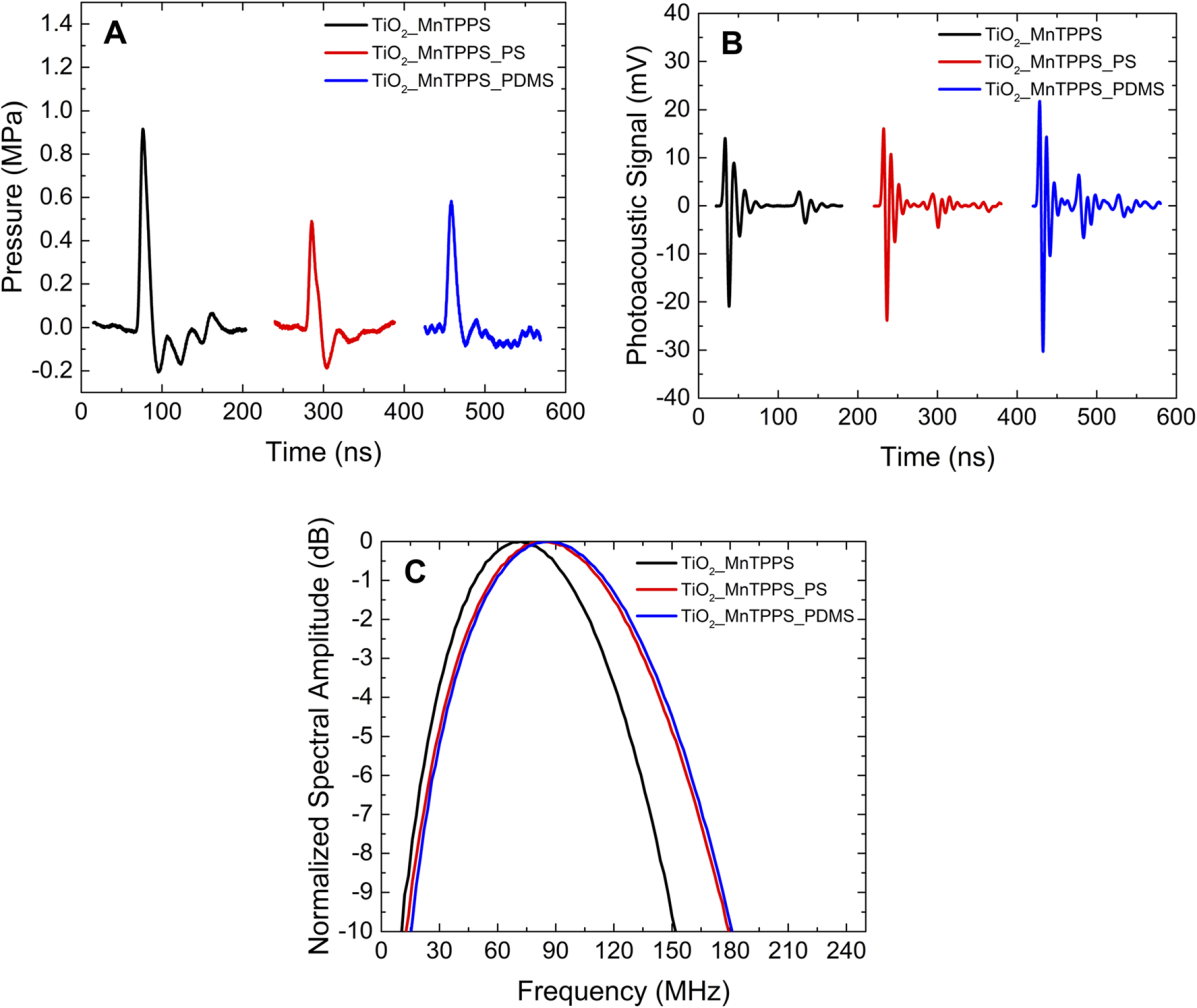
Characterization of piezophotonic materials at 471 nm using a 6 ns pulse laser (10 mJ cm^−2^): (A) absolute pressures using a 30 MHz needle hydrophone. The pressure transients were separated in time by 200 ns to avoid their overlapping; (B) main peaks of the photoacoustic waves detected by a 225 MHz contact transducer. The photoacoustic waves were separated in time by 200 ns to avoid their overlapping; (C) FFT treatment of the detected waves with the transducer.

The photoacoustic waves generated by TiO_2__MnTPPS, TiO_2__MnTPPS_PS, and TiO_2__MnTPPS_PDMS were obtained. We employed a Nd:YAG laser and OPO (FWHM 6 ns) with an energy fluence of 10 mJ cm^−2^ at 471 nm, where the absorption is >90% of the incident light, to generate the photoacoustic waves. The setup used is a front-face irradiation structure and the high frequencies were measured with a 225 MHz contact transducer as shown in Fig. 1. A small amount of silicone paste was applied as a coupling gel for TiO_2__MnTPPS, while water gel was used for the other samples. The peaks of the photoacoustic waves collected over an extended timescale are shown in Fig. 4B. The measurement of absolute pressure of the photoacoustic waves generated by the materials used a 30 MHz needle hydrophone and the setup is shown in Fig. 2. Fig. 4 shows the results obtained for each material in terms of absolute pressures and high-frequency distribution after FFT treatment of the photoacoustic waves.

**Fig. 5:**
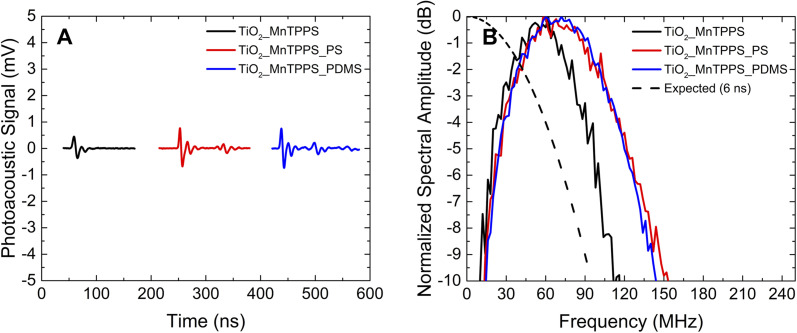
Photoacoustic signals obtained using a 6 ns pulse laser (2 mJ cm^−2^) at 532 nm: (A) main peaks of the photoacoustic waves detected with a 225 MHz contact transducer. The photoacoustic waves were separated in time by 200 ns to avoid their overlapping; (B) FFT treatment of the photoacoustic waves detected with the contact transducer. The dashed line represents the FFT of a Gaussian laser pulse with an FWHM of 6 ns.

**Fig. 6:**
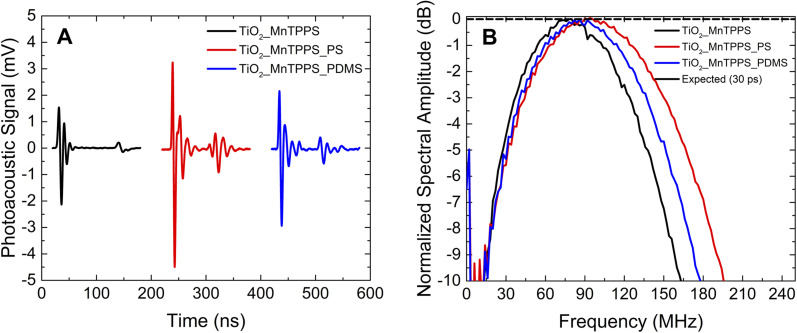
Photoacoustic signals obtained with the 30 ps pulse laser (2 mJ cm^−2^) at 532 nm: (A) main peaks of the photoacoustic waves detected with a 225 MHz contact transducer. The photoacoustic waves were separated in time by 200 ns to avoid their overlapping; (B) FFT treatment of the waves detected with the transducer. The dashed line represents the FFT of a Gaussian laser pulse with an FWHM of 30 ps.

We investigated the influence of the laser pulse duration on the photoacoustic wave using the same set of materials. We aimed to compare nanosecond and picosecond laser pulse widths in the generation of photoacoustic waves. Since our picosecond laser is limited to 532 nm, and considering the absorption spectrum of the materials, the studies were performed with excitation at this wavelength, using an energy fluence of 2 mJ cm^−2^. Fig. 5 and 6 show the photoacoustic waves and the frequency distribution obtained by FFT using a nanosecond laser (6 ns FWHM) and a picosecond laser (30 ps FWHM), respectively. These two figures share the same scales, in order to facilitate comparison.

For all the films and within the range of energy fluencies studied, a linear proportional relationship between the amplitude of the photoacoustic waves and the energy fluence was observed. Fig. S5 in the ESI† shows a graph of the maximum amplitude of the photoacoustic signal (measured by the 225 MHz contact transducer) as a function of the energy fluence within a range of 2 to 12 mJ cm^−2^ for the TiO_2__MnTPPS_PS film. The frequency distribution after FFT treatment of the photoacoustic waves was not affected by the energy fluences used (Fig. S5 in the ESI†). The non-proportional increase in the photoacoustic wave peak amplitudes as a function of applied energy fluence has been attributed to instrumental factors, degradation of the photoacoustic source, acoustic attenuation or non-linear propagation.^37^ No noticeable film degradation was observed during the experiments. To probe the performance under continuous laser exposure, the TiO_2__MnTPPS_PS and TiO_2__MnTPPS_PDMS films were submitted to 20 mJ cm^−2^ fluence pulses at 10 Hz for one hour and no change was observed in the amplitude of the photoacoustic waves (probed with a contact transducer; see Fig. S6, ESI†). The robustness of the films was quantified in terms of the laser-induced damage threshold. Although a film with MnTPPS adsorbed into TiO_2_ but without being permeated with silicone paste or polymer bleaches at relatively low laser fluence, the permeated films covered with polymers show laser-induced damage thresholds above ∼100 mJ cm^−2^ (see Table S2, ESI†).

## Discussion

The thickness and composition of each film were investigated by scanning electron microscopy (Fig. 3C). At the bottom of each image, it is possible to observe a dark region which corresponds to the glass slide where the material is deposited. Over the glass surface, there is a white region at the bottom of the film which corresponds to the TiO_2_ layer. Fig. 3(C1) corresponds to the film without any polymer, and it is possible to see the presence of the silicone paste which was used during the photoacoustic experiments. The film where we applied PS could be isolated from the glass slide, as shown in Fig. 3(C2), and the polymer is observed over the TiO_2_ layer. Furthermore, PS efficiently infiltrates the TiO_2_ mesoporous structure, which allows heat transfer from the absorber to the thermoelastic expansion material. No measurable photoacoustic waves were observed using TiO_2__MnTPPS films without infiltrated polymer or silicone paste when irradiated with a pulsed laser. Fig. 3(C3) illustrates the film with PDMS, in which the polymer layer is substantially thicker than the film with PS, because we used the same spin coating rotation for both cases, with PDMS having a higher viscosity than PS. In terms of absorption spectra, a well-defined Soret band is visible for MnTPPS in ethanol solution at nearly 466 nm with two less intense Q bands at 563 and 600 nm. Once adsorbed in the TiO_2_ structure the Soret band undergoes a redshift to 471 nm (Fig. 3B). Herein we use this wavelength, where the absorption is maximized, to excite the porphyrin molecules and generate photoacoustic waves. The large number of molecules of porphyrin present in a short space, allowed by the high surface area of the TiO_2_ nanoparticles, leads to materials with high linear absorption coefficients: a value of 523 mm^−1^ is achieved (excitation at 471 nm). The ultrathin TiO_2__MnTPPS absorption layer yields linear absorption coefficients comparable with those of the thinner carbon soot polymer composites produced,^25^ but significantly higher than thicker laser ultrasound transducers.^11^

The analysis of Fig. 4A shows that the photoacoustic waves have a profile that is mostly compressive, which is the result of an efficient confinement.^38^ For the film TiO_2__MnTPPS the absolute pressure obtained has an amplitude of 0.9 MPa. For those films which contain a polymer layer the amplitude is lower, with values of 0.5 and 0.6 MPa for TiO_2__MnTPPS_PS and TiO_2__MnTPPS_PDMS, respectively. For equivalent incident laser fluences the ultrasound pulse amplitude depends on light-to-sound conversion efficiency. As the three transducers share the same absorber molecule in equal quantity and within the same thickness, most likely the lower absolute pressure observed in films covered with a polymer layer results from the attenuation of the photoacoustic wave in this additional compact layer, while the viscous silicone paste allows virtually no additional layer to the 4.4 μm TiO_2_ film. The hypothesis of lower efficiency in heat transmission between the dye-coated TiO_2_ particles and the polymers compared to the silicone paste is contradictory, with the same range of frequencies obtained by the three films (see Fig. 6). The photoacoustic signals obtained with a broader contact transducer shown in Fig. 4B are higher for those films with PS or PDMS applied by spin coating. So, it is also possible that the absolute pressure results are underrated since the sensitivity of the hydrophone does not integrate the higher frequencies. The frequency distribution (Fig. 4C) is very similar for these two materials and reveals higher values of central frequency and larger bandwidths for the films with a polymer layer (indicative of slightly higher attenuation of high frequencies by the silicone paste). Even so, minimizing photoacoustic attenuation by using ultrathin photoacoustic sources allows upper high-frequency limits to be reached. Remarkable bandwidths of 133 MHz at −6 dB, a central frequency of ∼90 MHz (around 160 MHz for the upper bandwidth limit at −6 dB) for TiO_2__MnTPPS_PS and TiO_2__MnTPPS_PDMS were obtained.

There are examples of ultrathin films (less than 8 μm in thickness) used for ultrasound generation using nanosecond laser sources for irradiation. Although it is worth carrying out a comparative analysis, it should be considered that geometric factors (such as the distance at which the measurement is taken) or instrumental factors (as the type of detector and its frequency range) influence the measured values. Baac *et al.*^6^ reported ∼80 MHz bandwidth at −6 dB for 2.6 μm carbon nanotubes/PDMS composites (6 ns FWHM), whereas in the present work values in the order of 130 MHz were obtained. Chen *et al.*^39^ measured a very high energy conversion efficiency (8.3 × 10^−3^ with 45 mJ cm^−2^, 6 ns FWHM) for a multilayered carbon nanotube yarn PDMS composite, reaching a central frequency of 9.1 MHz and a maximum bandwidth of ∼30 MHz, in contrast with the central frequencies of ∼90 MHz and a maximum bandwidth of 133 MHz observed in this work using a 6 ns pulse. A carbon soot nanoparticle composite with PDMS with an absorbing layer of 6 μm yielded an energy conversion coefficient of 4.41 × 10^−3^ and −6 dB frequency bandwidth of 21 MHz (86 ns laser pulse).^25^ An ultrathin metal (10 nm, Cr) film sandwiched by polymer layers of less than 1 μm was fabricated by Lee and Guo.^3^ These authors do not mention the central frequency and bandwidth obtained but observed a pressure maximum of 1.8 MPa (with a 6 ns FWHM and low fluence of 2.23 mJ cm^−2^) that are higher than the 0.5 to 0.9 MPa (10 mJ cm^−2^) we observed with the TiO_2__MnTPPS films. Recently, a lead halide perovskite ultrathin layer (323 nm) sandwiched between two PDMS layers was proposed for efficient photoacoustic conversion, simultaneously achieving broad bandwidths (−6 dB bandwidth: 40.8 MHz, central frequency: 29.2 MHz), and high conversion efficiency (2.97 × 10^−2^).^40^ Although reaching high frequency and bandwidth with ultrathin films of MnTPPS adsorbed into TiO_2_, we obtained relatively low-pressure peaks (that are naturally scalable by increasing laser energy density) and low energy conversion efficiencies (order of 10^−5^).

High peak pressures of photoacoustic waves are important in the increase of signal-to-noise ratios for high-resolution imaging, but higher frequency components are important in improving image resolution or application towards the permeabilization of biological membranes. It was experimentally observed that the pressure waves, stress gradient and impulse are more relevant in cell permeabilization, than solely the peak pressure values.^41–43^ From the time profiles of the absolute pressure (Fig. 4A) stress gradients and impulses can be calculated: the stress gradient calculated as a peak pressure divided by the time from 10% to 90% of the peak pressure, and impulse calculated as the pressure integrated over the time of the compressional wave.^17^ The use of fast and low fluence laser pulses limits the impulse values achievable in our experiments, but stress gradients can be evaluated. Values of the stress gradient of 0.19, 0.09 and 0.1 MPa ns^−1^ were obtained for the TiO_2__MnTPPS films with silicone paste, PS and PDMS, respectively. Adsorbing MnTPPS on TiO_2_ in an ultrathin layer permits rather high-stress gradients: using a film of MnTPP dispersed in PS, lower values of 0.025 MPa ns^−1^ were obtained using a nanoseconds laser source; and even using picosecond laser sources, that should generate higher frequencies that favour the production of high-stress gradients, a lower value of 0.036 MPa ns^−1^ was observed.^17^

The results obtained by irradiation by nanosecond pulses may be compromised by the laser pulse width, since the photoacoustic generation occurs in the long-pulse regime. An efficient photoacoustic conversion requires both thermal and stress confinement. The nanosecond laser pulse may not ensure proper optical confinement, and to some extent thermal expansions may occur during optical heating. In order to compare the behavior of nanosecond *vs.* picosecond pulses we used 532 nm laser pulses of 6 ns and 30 ps. For the films under study the linear absorption coefficient is equal to 136 mm^−1^ at 532 nm. The speed of sound is 2400 m s^−1^ for films with PS and 1100 m s^−1^ for PDMS, and the values of thermal diffusivity are 2.0 × 10^−7^ and 1.1 × 10^−7^ m^2^ s^−1^,^2,10^ respectively. So, it leads to *τ*_th_ = 270 μs and *τ*_s_ = 3.1 ns for TiO_2__MnTPPS_PS and *τ*_th_ = 492.5 μs and *τ*_*s*_ = 6.7 ns for TiO_2__MnTPPS_PDMS. Thermal confinement is always established, but stress confinement is verified only by using a picosecond pulsed laser. So we may consider that the short-pulse conditions are not met when using nanosecond pulses, which indicates that the temporal profile of the photoacoustic pulse is limited by the laser pulse width.^32^

The ultrathin TiO_2__MnTPPS materials allow the influence of the laser pulse width on the shape of the observed photoacoustic waves to be studied: 30 ps pulse irradiation gives rise to sharper photoacoustic waves (comparison between Fig. 5A and 6A). The highest power of the laser pulses used for 30 ps irradiation leads to higher amplitude photoacoustic waves. Observed bandwidths and central frequencies are higher for 30 ps pulse excitation. The TiO_2__MnTPP_PS film yields a bandwidth (−6 dB) of 108 MHz for 6 ns pulse irradiation; enhanced to 148 MHz for 30 ps pulse irradiation. For the same film the upper frequency at −6 dB is improved from 127 MHz to 173 MHz. In addition to a broadening of the bandwidths for shorter laser pulses, there is also a general deviation of the central frequency to higher values. It can be noticed in Fig. 6B that the use of PS polymer as host leads to higher bandwidth and high frequencies compared with PDMS as a host. This may be attributed to the higher speed of sound of PS, which in practical terms reduces the thickness of the acoustic source.

Under the approximation that the laser pulses have a Gaussian shape, they can be described by the expression

5 *p*(*t*) = *p*_0_ exp[−4 ln(2)(*t*/*τ*_L_)^2^],

which enables an estimate by FFT treatment of the laser profile expected in the frequency-domain. Fig. 5 and 6 show the FFT of a Gaussian 6 ns and 30 ps laser pulse, respectively. By the analysis of Fig. 5 it is possible to observe remarkably large bandwidths even with 6 ns pulse irradiation. These are similar to those of the laser pulse with *t*_L_ = 6 ns, with bandwidth values of ∼100 MHz at −6 dB, for the TiO_2__MnTPPS_PS and TiO_2__MnTPPS_PDMS films. These results are very promising, but any enhancement will be limited by the laser pulse width, which determines the upper limit of achievable frequency band. This limitation can be lifted using pulses of shorter duration, as illustrated in Fig. 6B by the FFT of a Gaussian 30 ps pulse. The experiments with a 30 ps pulse laser are in the short-pulse regime and the maximum spectral photoacoustic wave is determined by the dimensions of the acoustic source and by the speed of sound, *i.e.*, *f* = *μ*_a_ × *c*_s_ = 1/*τ*_s_.^44^Fig. 6 shows that the bandwidths become ∼150 MHz at −6 dB and ∼170 MHz at −10 dB, for the TiO_2__MnTPPS_PS film, which are consistent with *f* = 326 MHz (the frequency spectrum of the contact transducer is presented in Fig. S7†).

To achieve high-frequency ultrasound we considered both laser pulse duration and the thickness of the acoustic source.^32^ Ultrafast heat deposition by MnTPPS and fast heat transfer to polymers, due to the nanoscale dimensions of the TiO_2_ particles, contribute to the efficient generation of high-frequency ultrasound. The reduced thickness of the absorbing and acoustic source layer, minimizing photoacoustic attenuation, allows upper high-frequency limits to be reached.^26^ The use of lasers with picosecond pulse durations allows the generation of the photoacoustic wave to depend only on the intrinsic properties of the materials.

The use of the photoacoustic mechanism in biomedical applications of ultrasound often relies on miniaturization and use of optical fibers.^8,9^ Coating of TiO_2_ on optical fibers is possible and has been done for applications such as photocatalytic reactors or sensors.^45,46^ Sophisticated sputtering techniques or simple dip-coating methods can be used to obtain uniform nano- to micrometer layers of TiO_2_ over the tip (and/or the sides) of optical fibers, in which the MnTPPS dye can adsorb at room temperature and be coated with a polymer layer.

A reduced laser-induced damage threshold could limit the applications of the developed piezophotonic materials. From our observations we can state that a remarkable property of the TiO_2_/MnTPPS/polymer films is that they can be used as piezophotonic materials for 36 000 laser pulses at laser fluences of 20 mJ cm^−2^ for 30 ps pulses (0.7 GW cm^−2^), without visible degradation or change in photoacoustic properties. Although a TiO_2__MnTPPS film without being permeated with silicone paste or polymer bleaches at relatively low laser fluence, the films with polymer coating show laser-induced damage thresholds above ∼100 mJ cm^−2^ (ESI, Table S2†), most probably due to the fast heat transfer permitted by the polymer presence. Although the damage mechanism is distinct (bleaching *vs.* ablation) the values obtained are within the same range of values reported for carbon soot nanoparticle based piezophotonic materials (*e.g.* 81 mJ cm^−2^,^47^ 108.6 mJ cm^−2^ (ref. 48)).

## Conclusions

Pulsed laser irradiation of ultrathin films consisting of a layer of TiO_2_ nanoparticles (4.4 μm) on which a manganese porphyrin dye with an ultra-fast and practically unitary quantum yield of non-radiative decay is adsorbed, embedded in polymers with a convenient Grüneisen coefficient (silicone paste, polystyrene and polydimethylsiloxane), gives rise to wide bandwidth ultrasound, ∼150 MHz at −6 dB. Steep stress gradients of 0.19 MPa ns^−1^ are obtained. The use of lasers with pulse durations of 30 ps overcomes limitations associated with ns pulse duration lasers, allowing the generation of the photoacoustic wave to depend only on the intrinsic properties of the materials. We show that remarkably high frequency is achieved, ∼170 MHz at −6 dB, when a combination of low-thickness acoustic source and short-pulsed lasers are used. Photoacoustic waves with high-frequency components like the ones obtained here are relevant for bioimaging applications, improving imaging resolution. In soft tissues high frequency ultrasound waves, like the ones obtained with the piezophotonic materials presented here, are strongly attenuated for clinically relevant path lengths. For imaging applications this leads to a trade-off between resolution and imaging depth. Considering the bandwidth and central frequency of the ultrasound pulses obtained axial resolutions of 10 μm at depths less than 1 mm can be achieved. The steep stress gradients obtained should be appropriate to the permeabilization of biological membranes toward drug delivery.

## Author contributions

C. S. conceived the idea and methodology. S. M. A. P. synthesized the dye. T. B. P. prepared the materials and conducted the experimental photoacoustic characterization. A. P. P. characterized the materials. All authors contributed to the analysis of the data. T. B. P and C. S. wrote the manuscript.

## Conflicts of interest

The authors declare that they have no known competing financial interests or personal relationships that could have appeared to influence the work reported in this paper.

## Supplementary Material

NA-005-D3NA00451A-s001

## References

[cit1] Kim J., Kim H., Chang W. Y., Huang W., Jiang X., Dayton P. A. (2019). Candle-Soot Carbon Nanoparticles in Photoacoustics: Advantages and Challenges for Laser Ultrasound Transmitters. IEEE Nanotechnol. Mag..

[cit2] Noimark S., Colchester R. J., Poduval R. K., Maneas E., Alles E. J., Zhao T., Zhang E. Z., Ashworth M., Tsolaki E., Chester A. H., Latif N., Bertazzo S., David A. L., Ourselin S., Beard P. C., Parkin I. P., Papakonstantinou I., Desjardins A. E. (2018). Polydimethylsiloxane Composites for Optical Ultrasound Generation and Multimodality Imaging. Adv. Funct. Mater..

[cit3] Lee T., Guo L. J. (2017). Highly Efficient Photoacoustic Conversion by Facilitated Heat Transfer in Ultrathin Metal Film Sandwiched by Polymer Layers. Adv. Opt. Mater..

[cit4] Chang W. Y., Zhang X. A., Kim J., Huang W., Bagal A., Chang C. H., Fang T., Wu H. F., Jiang X. (2018). Evaluation of Photoacoustic Transduction Efficiency of Candle Soot Nanocomposite Transmitters. IEEE Trans. Nanotechnol..

[cit5] Lee T., Baac H. W., Ok J. G., Jay Guo L. (2018). Polymer-Nanomaterial Composites for Optoacoustic Conversion. Funct. Org. Hybrid Nanostruct. Mater..

[cit6] Won Baac H., Ok J. G., Park H. J., Ling T., Chen S. L., Hart A. J., Guo L. J. (2010). Carbon nanotube composite optoacoustic transmitters for strong and high frequency ultrasound generation. Appl. Phys. Lett..

[cit7] Mason T. J. , Bernal V. S., An introduction to sonoelectrochemistry, Power Ultrasound in Electrochemistry: From Versatile Laboratory Tool to Engineering Solution, 2012, pp. 21–44, 10.1002/9781119967392

[cit8] Li J., Ma Y., Zhang T., Shung K. K., Zhu B. (2022). Recent Advancements in Ultrasound Transducer: From Material Strategies to Biomedical Applications. BME Front..

[cit9] Barbosa R. C. S., Mendes P. M. (2022). A Comprehensive Review on Photoacoustic-Based Devices for Biomedical Applications. Sensors.

[cit10] Silva A. D., Henriques C. A., V Malva D., Calvete M. J. F., Pereira M. M., Serpa C., Arnaut L. G. (2020). Photoacoustic generation of intense and broadband ultrasound pulses with functionalized carbon nanotubes. Nanoscale.

[cit11] Sá G. F. F., Serpa C., Arnaut L. G. (2013). Stratum corneum permeabilization with photoacoustic waves generated by piezophotonic materials. J. Controlled Release.

[cit12] Hou Y., Ashkenazi S., Huang S. W., O'Donnell M. (2008). An integrated optoacoustic transducer combining etalon and black PDMS structures. IEEE Trans. Ultrason. Ferroelectr. Freq. Control..

[cit13] Wang S., Echeverry J., Trevisi L., Prather K., Xiang L., Liu Y. (2020). Ultrahigh resolution pulsed laser-induced photoacoustic detection of multi-scale damage in CFRP composites. Appl. Sci..

[cit14] Guggenheim J. A., Zhang E. Z., Beard P. C. (2017). A Method for Measuring the Directional Response of Ultrasound Receivers in the Range Ultrasound Source. IEEE Trans. Ultrason. Ferroelectr. Freq. Control..

[cit15] Rajagopal S., Cox B. T. (2020). 100 MHz bandwidth planar laser-generated ultrasound source for hydrophone calibration. Ultrasonics.

[cit16] Henriques J., Schaberle F. A., Serpa C., Pais A. A. C. C., Cardoso C., Vitorino C. (2021). Photoacoustic method for real-time assessment of salt content in aqueous solutions. Talanta.

[cit17] Silva A. D., Serpa C., Arnaut L. G. (2019). Photoacoustic transfection of DNA encoding GFP. Sci. Rep..

[cit18] Pereira D. A., Silva A. D., Martins P. A. T., Piedade A. P., Martynowych D., Veysset D., Moreno M. J., Serpa C., Nelson K. A., Arnaut L. G. (2021). Imaging of photoacoustic-mediated permeabilization of giant unilamellar vesicles (GUVs). Sci. Rep..

[cit19] Buma T., Spisar M., O'Donnell M. (2001). High-frequency ultrasound array element using thermoelastic expansion in an elastomeric film. Appl. Phys. Lett..

[cit20] Baac H. W., Ling T., Ashkenazi S., Huang S.-W., Guo L. J. (2010). Photoacoustic concave transmitter for generating high frequency focused ultrasound. Photons Plus Ultrasound Imaging Sens..

[cit21] Colchester R. J., Mosse C. A., Bhachu D. S., Bear J. C., Carmalt C. J., Parkin I. P., Treeby B. E., Papakonstantinou I., Desjardins A. E. (2014). Laser-generated ultrasound with optical fibres using functionalised carbon nanotube composite coatings. Appl. Phys. Lett..

[cit22] Hsieh B. Y., Kim J., Zhu J., Li S., Zhang X., Jiang X. (2015). A laser ultrasound transducer using carbon nanofibers-polydimethylsiloxane composite thin film. Appl. Phys. Lett..

[cit23] Lee S. H., Park M., Yoh J. J., Song H., Yun E. (2012). Reduced graphene oxide coated thin aluminum film as an optoacoustic transmitter for high pressure and high frequency ultrasound generation. Appl. Phys. Lett..

[cit24] Vella D., Mrzel A., Drnovšek A., Shvalya V., Jezeršek M. (2022). Ultrasonic photoacoustic emitter of graphene-nanocomposites film on a flexible substrate. Photoacoustics.

[cit25] Chang W. Y., Huang W., Kim J., Li S., Jiang X. (2015). Candle soot nanoparticles-polydimethylsiloxane composites for laser ultrasound transducers. Appl. Phys. Lett..

[cit26] Hou Y., Kim J. S., Ashkenazi S., O’Donnell M., Guo L. J. (2006). Optical generation of high frequency ultrasound using two-dimensional gold nanostructure. Appl. Phys. Lett..

[cit27] Wu N., Tian Y., Zou X., Silva V., Chery A., Wang X. (2012). High-efficiency optical ultrasound generation using one-pot synthesized polydimethylsiloxane-gold nanoparticle nanocomposite. J. Opt. Soc. Am. B.

[cit28] Karabutov A. A., Savateeva E. V., Podymova N. B., Oraevsky A. A. (2000). Backward mode detection of laser-induced wide-band ultrasonic transients with optoacoustic transducer. J. Appl. Phys..

[cit29] Schaberle F. A., Nunes R. M. D., Barroso M., Serpa C., Arnaut L. G. (2010). Analytical solution for time-resolved photoacoustic calorimetry data and applications to two typical photoreactions. Photochem. Photobiol. Sci..

[cit30] Arnaut L. G., Caldwell R. A., Elbert J. E., Melton L. A. (1992). Recent advances in photoacoustic calorimetry: Theoretical basis and improvements in experimental design. Rev. Sci. Instrum..

[cit31] Wang L. V. (2008). Photoacoustic microscopy and computed tomography. Biomed. Opt. BIOMED.

[cit32] Lee T., Baac H. W., Li Q., Guo L. J. (2018). Efficient Photoacoustic Conversion in Optical Nanomaterials and Composites. Adv. Opt. Mater..

[cit33] Pinto S. M. A., Calvete M. J. F., Ghica M. E., Soler S., Gallardo I., Pallier A., Laranjo M. B., Cardoso A. M. S., Castro M. M. C. A., Brett C. M. A., Pereira M. M., Tóth É., Geraldes C. F. G. C. (2019). A biocompatible redox MRI probe based on a Mn(ii)/Mn(iii) porphyrin. Dalton Trans..

[cit34] Schaberle F. A., Abreu A. R., Gonçalves N. P. F., Sá G. F. F., Pereira M. M., Arnaut L. G. (2017). Ultrafast Dynamics of Manganese(III), Manganese(II), and Free-Base Bacteriochlorin: Is There Time for Photochemistry?. Inorg. Chem..

[cit35] Yan X., Kirmaier C., Holten D. (1988). A picosecond study of rapid multistep radiationless decay in manganese(III) porphyrins. Inorg. Chem..

[cit36] Irvine M. P., Harrison R. J., Strahand M. A., Beddard G. S. (1985). Picosecond Spectroscopy and Kinetics of Metalloporphyrins. Ber. Bunsenges. Phys. Chem..

[cit37] Rajagopal S., Sainsbury T., Treeby B. E., Cox B. T. (2018). Laser generated ultrasound sources using carbon-polymer nanocomposites for high frequency metrology. J. Acoust. Soc. Am..

[cit38] Carome E. F., Clark N. A., Moeller C. E. (1964). Generation of acoustic signals in liquids by ruby laser-induced thermal stress transients. Appl. Phys. Lett..

[cit39] Chen Z., Wu Y., Yang Y., Li J., Xie B., Li X., Lei S., Ou-Yang J., Yang X., Zhou Q., Zhu B. (2018). Multilayered carbon nanotube yarn based optoacoustic transducer with high energy conversion efficiency for ultrasound application. Nano Energy.

[cit40] Du X., Li J., Niu G., Yuan J. H., Xue K. H., Xia M., Pan W., Yang X., Zhu B., Tang J. (2021). Lead halide perovskite for efficient optoacoustic conversion and application toward high-resolution ultrasound imaging. Nat. Commun..

[cit41] Ando T., Sato S., Ashida H., Obara M. (2013). Effects of pressure characteristics on transfection efficiency in laser-induced stress wave-mediated gene delivery. Appl. Phys. A: Mater. Sci. Process..

[cit42] Kodama T., Hamblin M. R., Doukas A. G. (2000). Cytoplasmic molecular delivery with shock waves: Importance of impulse. Biophys. J..

[cit43] Doukas A. G., McAuliffe D. J., Lee S., Venugopalan V., Flotte T. J. (1995). Physical factors involved in stress-wave-induced cell injury: The effect of stress gradient. Ultrasound Med. Biol..

[cit44] Esenaliev R. O., Alma H., Tittel F. K., Oraevsky A. A. (1998). Axial resolution of laser opto-acoustic imaging: influence of acoustic attenuation and diffraction. Laser-Tissue Interact. IX.

[cit45] Harrisankar N., Levecque P., van Steen E. (2021). An automated coating process to produce TiO_2_-coated optical fibre for photocatalytic reactor systems. Chem. Eng. Process. - Process Intensif..

[cit46] Silva D., Monteiro C. S., Silva S. O., Frazão O., Pinto J. V., Raposo M., Ribeiro P. A., Sério S. (2022). Sputtering Deposition of TiO_2_ Thin Film Coatings for Fiber Optic Sensors. Photonics.

[cit47] Li Y., Guo Z., Li G., Chen S.-L. (2018). Miniature fiber-optic high-intensity focused ultrasound device using a candle soot nanoparticles-polydimethylsiloxane composites-coated photoacoustic lens. Opt. Express.

[cit48] Faraz M., Abbasi M. A., Sang P., Son D., Baac H. W. (2020). Stretchable and robust candle-soot nanoparticle-polydimethylsiloxane composite films for laser-ultrasound transmitters. Micromachines.

