# Supplementary material for: Ultrathin materials for wide bandwidth laser ultrasound generation: titanium dioxide nanoparticle films with adsorbed dye
Source: Nanoscale Adv. 2023 Jul 17;5(16):4191–202. doi: 10.1039/d3na00451a (PMC10408605; doi:10.1039/d3na00451a)
Supplement: NA-005-D3NA00451A-s001 [file NA-005-D3NA00451A-s001.pdf]

Electronic Supplementary Material (ESI) for Nanoscale Advances.
This journal is © The Royal Society of Chemistry 2023

Supplementary Information

Ultrathin materials for wide bandwidth laser ultrasound generation: titanium dioxide nanoparticles films with adsorbed dye

Tiago B. Pinto,[a] Sara M. A. Pinto,[a] Ana P. Piedade,[b] Carlos Serpa[a*]

[a]CQC-IMS, Department of Chemistry, University of Coimbra, 3004-535 Coimbra, Portugal.

[b]CEMMPRE, Department of Mechanical Engineering, University of Coimbra, 3030-788 Coimbra, Portugal.

[*]Corresponding author.

E-mail address: serpasoa@ci.uc.pt (C. Serpa)

Supplementary Fig. S1: Synthesis of the TPPS molecule. The 5,10,15,20-tetrakis(4-sulfonylphenyl)porphyrin molecule of manganese(III) acetate was synthesized in the laboratory of Catalysis and Fine Chemistry of the Chemistry Center of the University of Coimbra. MnTPPS is synthesized from the condensation of pyrrole with benzaldehyde in acetic acid and nitrobenzene in a 7:3 portion, followed by chlorosulfonation in which chlorosulfonic acids are added in excess to the TPP molecule and terminated with hydrolysis. ------------------------2
Supplementary Fig. S2: Synthesis of MnTPPS, with the introduction of manganese (III) metal. The introduction of the transition metal takes place by adding an excess of manganese acetate tetrahydrate in sodium acetate/acetic acid, followed by purification by a size exclusion column chromatography. --3
Supplementary Fig. S3: Photoacoustic waves generated by piezophotonic materials of equal absorption and different thickness: (Left) Photoacoustic signal collected; (Right) maximum amplitude of the photoacoustic wave as a function of the reciprocal of the material thickness. ---5
Supplementary Fig. S4: (Left) Frequency distributions of the photoacoustic waves of piezophotonic materials TiO_2_A-E; (Right) Central frequency dependence (red circle), bandwidth at -6 dB (blue square) and maximum bandwidth value at -6 dB as a function of the reciprocal of thickness. ---5
Supplementary Fig. S5: (Left) Graphic representation of the photoacoustic signal corresponding to the maximum amplitude as a function of energy fluency, with a 6 ns laser for TiO2_MnTPPS_PS; (Right) Frequency distribution after FFT treatment for various energy fluences with a 6 ns laser for TiO2_MnTPPS_PS. ------------------------------6
Supplementary Fig. S6: TiO_2_MnTPPS_PS (left) and TiO_2_MnTPPS_PDMS (right) film performance under continuous laser exposure for one hour (20 mJ cm^{-2} fluence pulses, 30 ps pulse width, 10 Hz). The graphs show normalized peak-to-peak amplitudes of the photoacoustic waves probed by a 225 MHz contact transducer before and every 15 minutes after starting laser exposure. The films did not show any visible degradation. -----------------7
Supplementary Fig. S7: Frequency spectrum of the 225 MHz V2113 Olympus contact transducer (serial number 871543). Central frequency: 203 MHz, Peak frequency: 155 MHz, -6 dB: [88 MHz to 318 MHz]. ------------------8
Supplementary Table S1: Properties of piezophotonic materials used to study the influence of thickness on the photoacoustic wave; [a]μ_a = $2.3A/l$, Napierian absorption coefficient. Films TiO_2_MnTPPS with silicone paste as expanding and coupling media. 4
Supplementary Table S2: Photographs showing the films surface after pulsed laser irradiation (pulse width: 30 ps, diameter 4 mm). Laser fluence is denoted at the second row. Laser ablation of the TiO_2_MnTPPS film (without silicone paste) starts to appear with the fluence of 60 mJ cm^{-2}. For both the polymer coated TiO_2_MnTPPS_PS and TiO_2_MnTPPS_PDMS a slight discoloration starts to appear at 100 mJ cm^{-2} and it is better noticed at 120 mJ cm^{-2}. In the first film (TiO_2_MnTPPS) the absence of heat dissipation in the polymer leads to early bleaching. 8

MnTPPS Synthesis

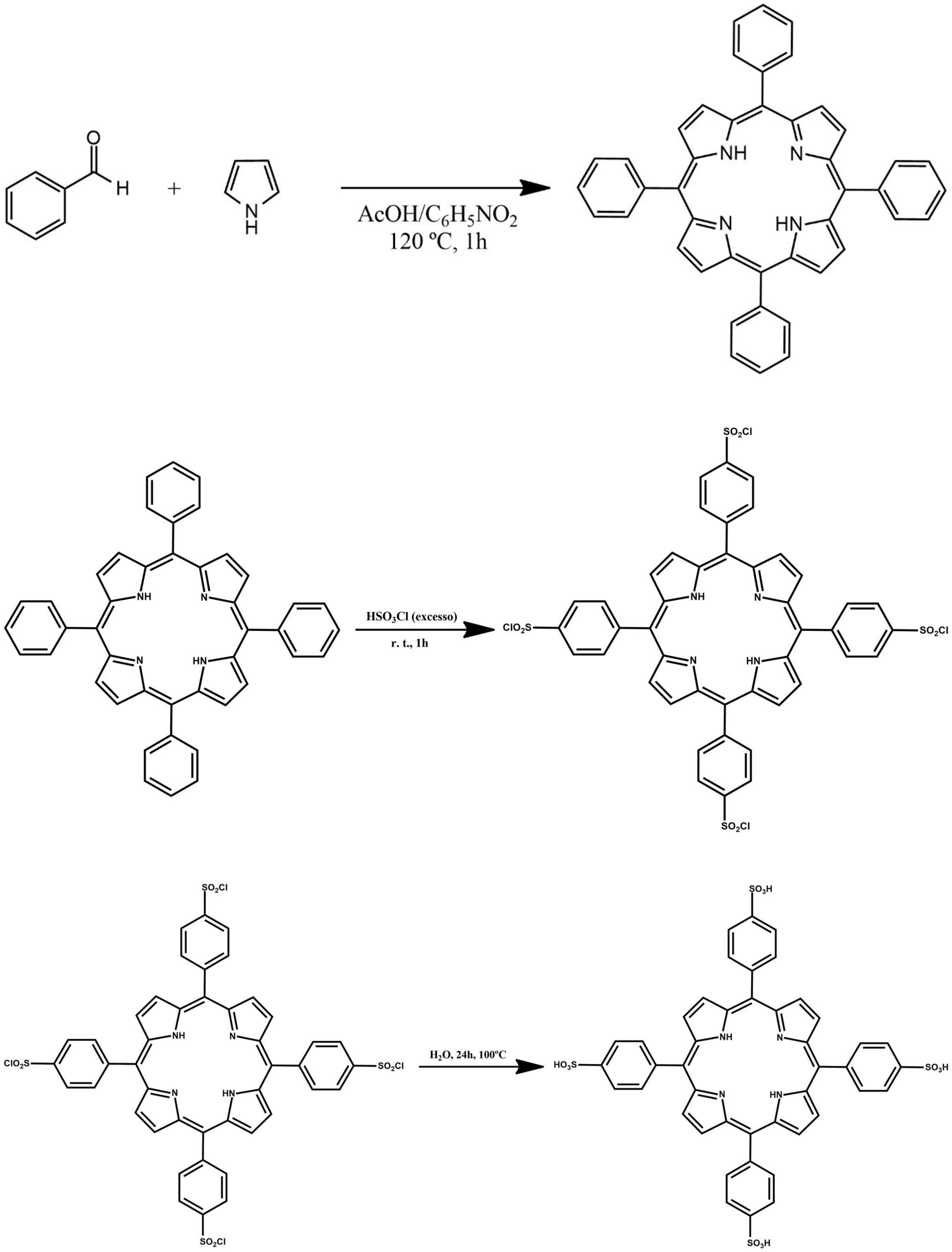

Supplementary Fig. S1: Synthesis of the TPPS molecule. The 5,10,15,20-tetrakis(4-sulfonylphenyl)porphyrin molecule of manganese(III) acetate was synthesized in the laboratory of Catalysis and Fine Chemistry of the Chemistry Center of the University of Coimbra. MnTPPS is synthesized from the condensation of pyrrole with benzaldehyde in acetic acid and

nitrobenzene in a 7:3 portion, followed by chlorosulfonation in which chlorosulfonic acids are added in excess to the TPP molecule and terminated with hydrolysis.

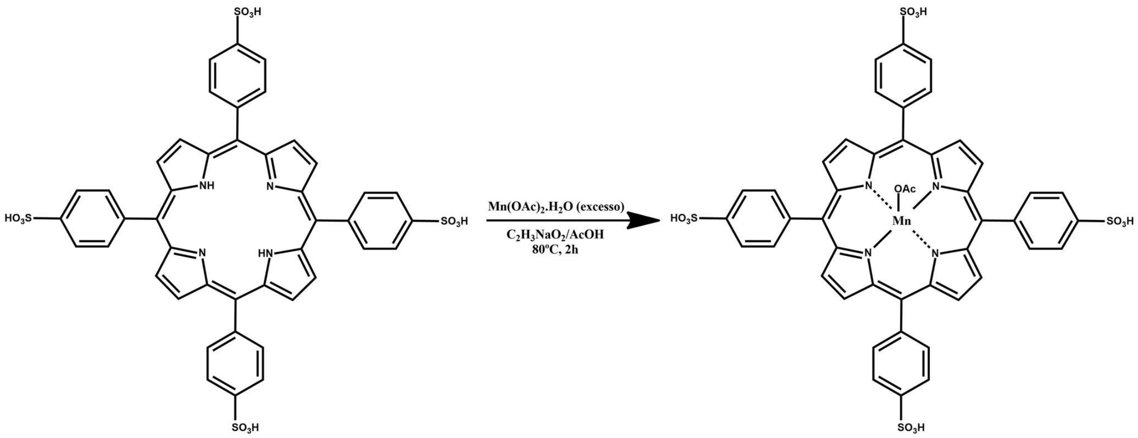

Supplementary Fig. S2: Synthesis of MnTPPS, with the introduction of manganese (III) metal. The introduction of the transition metal takes place by adding an excess of manganese acetate tetrahydrate in sodium acetate/acetic acid, followed by purification by a size exclusion column chromatography.

Influence of thickness on the photoacoustic wave

The influence of the thickness on the photoacoustic performance was studied. We prepared TiO_2 films with thicknesses of 3.5, 4.4 and 5.3 µm by screen-printing and 5.5 and 7.0 µm using doctor blade techniques. The thickness of each film was confirmed by SEM. The absorption was matched to 0.6 at the wavelength of 471 nm, which is the maximum absorption of the Soret band. The Supplementary Table S1 has the parameters of thickness, absorbance and linear absorption coefficient for each film.

Supplementary Table S1: Properties of piezophotonic materials used to study the influence of thickness on the photoacoustic wave; $^a\mu_a = 2.3A/l$, Napierian absorption coefficient. Films TiO_2_MnTPPS with silicone paste as expanding and coupling media.

Film	Thickness (µm)	Absorbance at 471 nm	$\mu_a{}^a$ (mm^{-1})
TiO2_A	3.5	0.6	394
TiO2_B	4.4	0.6	314
TiO2_C	5.3	0.6	260
TiO2_D	5.5	0.6	251
TiO2_E	7.0	0.6	197

We employed a nanosecond Nd:YAG laser (EKSPLA OPO model PG-122 pumped by an EKSPLA NL301G Nd:YAG laser delivering 6 ns), with excitation at 471 nm and an energy fluence of 10 mJ cm^{-2}. The high frequencies were studied by a 225 MHz transducer, and we used a front-face irradiation setup. Fig. S3 and S4 show the behavior of the photoacoustic signal and frequency distribution for different material thicknesses.

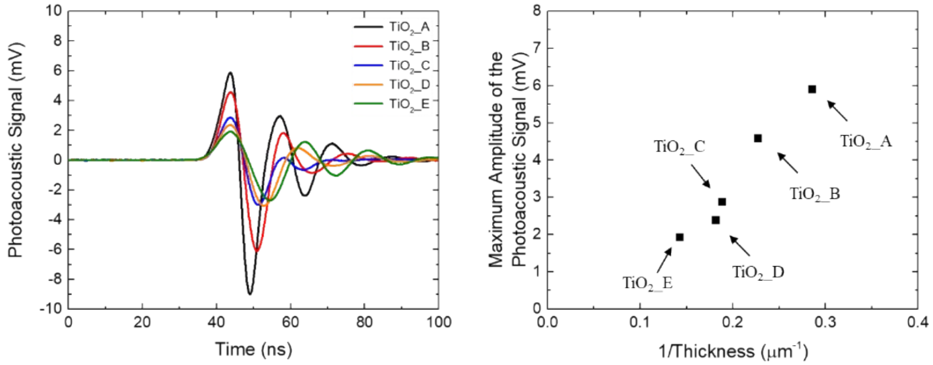

Supplementary Fig. S3: Photoacoustic waves generated by piezophotonic materials of equal absorption and different thickness: (Left) Photoacoustic signal collected; (Right) maximum amplitude of the photoacoustic wave as a function of the reciprocal of the material thickness.

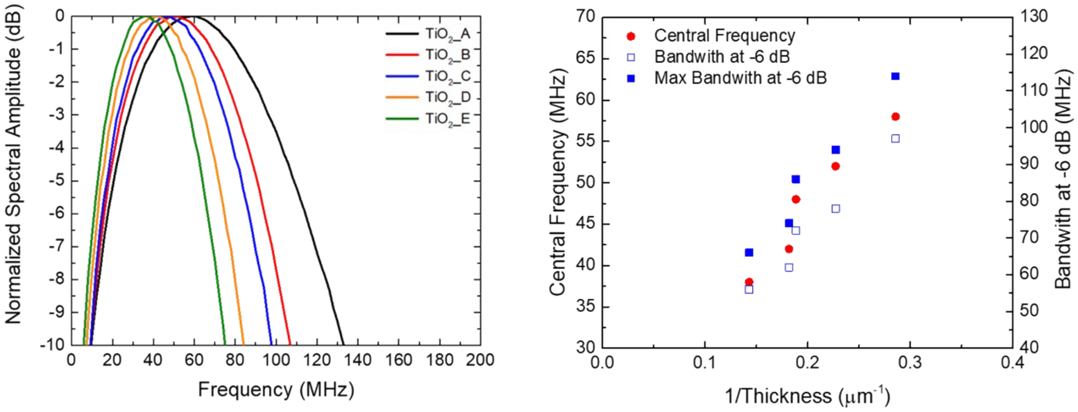

Supplementary Fig. S4: (Left) Frequency distributions of the photoacoustic waves of piezophotonic materials TiO_2_A-E; (Right) Central frequency dependence (red circle), bandwidth at -6 dB (blue square) and maximum bandwidth value at -6 dB as a function of the reciprocal of thickness.

Influence of energy on photoacoustic wave generation

By the equation (2) the intensity of the photoacoustic wave is proportional to the quantity of energy applied to the piezophotonic material. We were interested in understanding if the frequency distribution profile changes with the energy in a range of energy used in the previous experiments. We used a filter on the front of the laser beam since our nanosecond does not allow to change the energy manually. The photoacoustic waves were generated by a nanosecond Nd:YAG laser (EKSPLA OPO model PG-122 pumped by an EKSPLA NL301G Nd:YAG laser delivering 4–6 ns), with excitation at 471 nm and using a front-face irradiation setup. The energy was measured before each experiment with a Power Meter (Model 1918-C). Figure S5 shows a graph of the maximum amplitude of the photoacoustic signal in function to the energy fluence within a range of 2 to 12 mJ cm-2 for TiO2_MnTPPS_PS film.

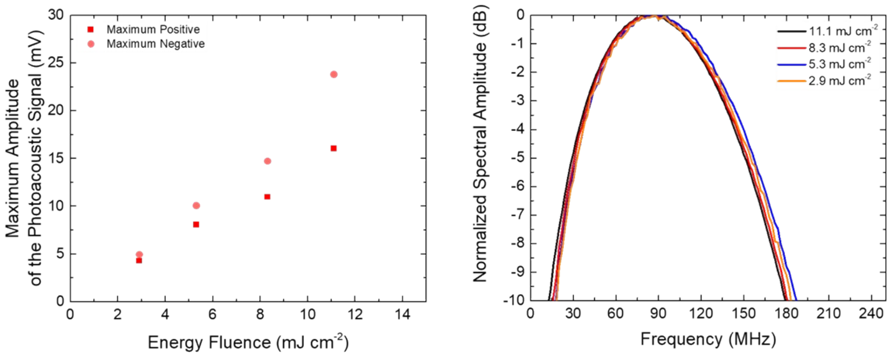

Supplementary Fig. S5: (Left) Graphic representation of the photoacoustic signal corresponding to the maximum amplitude as a function of energy fluency, with a 6 ns laser for TiO2_MnTPPS_PS; (Right) Frequency distribution after FFT treatment for various energy fluences with a 6 ns laser for TiO2_MnTPPS_PS.

Laser damage thresholds and performance under continuous laser exposure

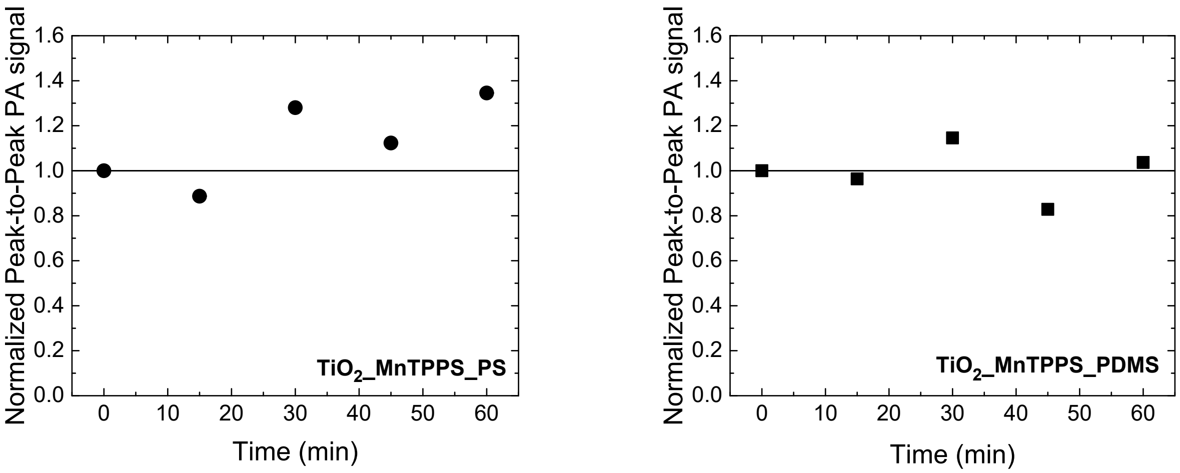

Supplementary Fig. S6: TiO_2_MnTPPS_PS (left) and TiO_2_MnTPPS_PDMS (right) film performance under continuous laser exposure for one hour (20 mJ cm^{-2} fluence pulses, 30 ps pulse width, 10 Hz). The graphs show normalized peak-to-peak amplitudes of the photoacoustic waves probed by a 225 MHz contact transducer before and every 15 minutes after starting laser exposure. The films did not show any visible degradation.

Supplementary Table S2: Photographs showing the films surface after pulsed laser irradiation (pulse width: 30 ps, diameter 4 mm). Laser fluence is denoted at the second row. Laser ablation of the TiO_2_MnTPPS film (without silicone paste) starts to appear with the fluence of 60 mJ cm^{-2}. For both the polymer coated TiO_2_MnTPPS_PS and TiO_2_MnTPPS_PDMS a slight discoloration starts to appear at 100 mJ cm^{-2} and it is better noticed at 120 mJ cm^{-2}. In the first film (TiO_2_MnTPPS) the absence of heat dissipation in the polymer leads to early bleaching.

	Fluence / mJ cm^{-1}						
Sample	0	20	40	60	80	100	120
TiO_2_MnTPPS (without silicone paste)	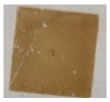	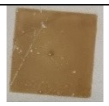	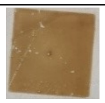	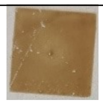	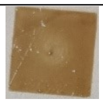	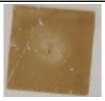	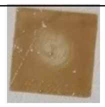
TiO_2_MnTPPS_PS	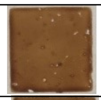	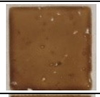	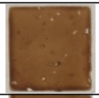	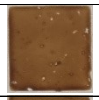	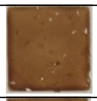	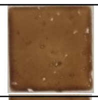	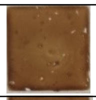
TiO_2_MnTPPS_PDMS	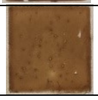	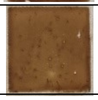	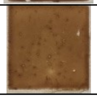	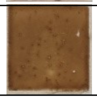	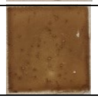	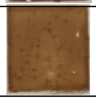	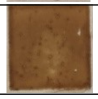

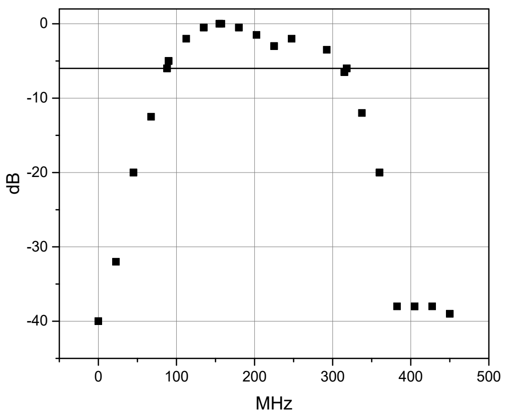

Supplementary Fig. S7: Frequency spectrum of the 225 MHz V2113 Olympus contact transducer (serial number 871543). Central frequency: 203 MHz, Peak frequency: 155 MHz, -6 dB: [88 MHz to 318 MHz].
